# TKS—The gentle *molder*

**Published:** 2008

**Authors:** Surendar Mohan Tuli

**Affiliations:** *VIMHANS, Lajpat Nagar New Delhi. E-mail: smohantuli@yahoo.co.in*

With the demise of TKS, I lost a personal friend. In our fruitful association of more than 45 years (1960–2008), we spent many hours together in educational programs, research meetings, selection committees, and professional conferences. We agreed to disagree in many aspects; however, we were in consonance in many more aspects of health care delivery for common orthopedic patients and orthopedic education. To this generation of orthopedic specialists, he frequently reminded that the most important first aid for a spinal cord injured patient is transportation from the site of accident on a simple padded board 6 feet × 2 ½ feet. He would lament loudly that less than 5% of such patients reach a hospital in less than 8 h of the trauma, and that 50% of cervical cord injured patients die within a few weeks because of quadriplegia. He developed, improvised, propagated, and achieved excellent results in the rehabilitation of spinal cord damaged patient with the use of three-pillow technique of nursing of patients. The upper and lower pillows to help log-rolling turning of the paralyzed patient with minimal help and the middle pillow placed at the site of vertebral column fracture as gentle molder while biological process of healing was in progress. He rightly questioned: do all patients benefit by aggressive and expensive surgeries in spinal cord injured patient? He often pleaded: do not increase the anguish of the family by depleting the scant resources of a common spinal cord injured patient. Offering operative treatment for selected patients who have some likelihood of neural recovery, which he rightly insisted, can be judged by repeated careful clinical examination. In nonoperative treatment, the family can share the burden of nursing. The best of medical graduates were attracted to pursue postgraduate courses in orthopedics under him. This gentle molder nurtured a breed of orthopedic surgeons who proved to be enquiring, empathetic, ethical, skillful, and humane doctors for the society. Any person who was trained under him or was associated with him had an experience that influenced their whole career. Throughout his professional and academic career, he had the rare talent of recognizing a good idea or subject for further workup. This resulted in a number of significant publications in the management of spinal cord injuries, tuberculosis of bones, joints and spine, medical education, and health care delivery system. According to the dictates of his conscience, he improvised many endeavors for doing good to the largest number.

He was a voracious reader to mold himself, a frequent lecturer to mold the opinion of the audience, and a prolific writer with a hope to sustain the molding of the thought of the next generations.

